# A Novel Ferroptosis-Related Gene Signature for Overall Survival Prediction in Patients With Breast Cancer

**DOI:** 10.3389/fcell.2021.670184

**Published:** 2021-06-17

**Authors:** Lizhe Zhu, Qi Tian, Siyuan Jiang, Huan Gao, Shibo Yu, Yudong Zhou, Yu Yan, Yu Ren, Jianjun He, Bin Wang

**Affiliations:** ^1^Department of Breast Surgery, The First Affiliated Hospital of Xi’an Jiaotong University, Xi’an, China; ^2^Department of Medical Oncology, The First Affiliated Hospital of Xi’an Jiaotong University, Xi’an, China

**Keywords:** breast cancer, ferroptosis, gene signature, overall survival, immune status

## Abstract

**Introduction:**

Breast cancer is the most common malignant tumor in women worldwide. However, advanced multidisciplinary therapy cannot rescue the mortality of high-risk breast cancer metastasis. Ferroptosis is a newly discovered form of regulating cell death that related to cancer treatment, especially in eradicating aggressive malignancies that are resistant to traditional therapies. However, the prognostic value of ferroptosis-related gene in breast cancer remains unknown.

**Materials and Methods:**

In this study, a total of 1,057 breast cancer RNA expression data with clinical and follow-up information were downloaded from the TCGA cohort, multivariate Cox regression was used to construct the 11-gene prognostic ferroptosis-related gene signature. The breast cancer patients from the GEO cohort were used for validation. The expression levels of core prognostic genes were also verified in erastin-treated breast cancer cell lines by real-time polymerase chain action (PCR).

**Results and Discussion:**

Our results showed that 78% ferroptosis-related genes were differentially expressed between breast cancer tumor tissue and adjacent non-tumorous tissues, including 29 of them which were significantly correlated with OS in the univariate Cox regression analysis. Patients were divided into high-risk group and low-risk group by the 11-gene signature. Patients with high-risk scores had a higher probability of death earlier than the low-risk group both in the TCGA construction cohort and in the GEO validation cohort (all *P* < 0.001). Meanwhile, the risk score was proved to be an independent predictor for OS in both univariate and multivariate Cox regression analyses (HR > 1, *P* < 0.01). The predictive efficacy of the prognostic signature for OS was further verified by the time-dependent ROC curves. Moreover, we also enriched many immune-related biological processes in later functional analysis; the immune status showed a statistical difference between the two risk groups. In addition, the differences in expression levels of 11 core prognostic genes were examined in ferroptosis inducer-treated breast cancer cell lines.

**Conclusion:**

In conclusion, a novel ferroptosis-related gene model can be used for prognostic prediction in breast cancer. New ferroptosis-related genes may be used for breast cancer targeting therapy in the future.

## Introduction

Breast cancer is the most common malignant tumor in women worldwide; among 70–80% patients with early, non-metastatic disease can be cured. However, advanced breast cancer with distant organ metastasis is considered to be incurable by the currently available treatment methods (Harbeck et al., 2019). Meanwhile, the chemotherapy or endocrine therapy resistance of breast cancer patients also brings certain challenges to their treatment. In addition, the global incidence of breast cancer is increasing at a rate of 3.1% per year, from 641,000 cases in 1980 to 1.6 million cases in 2010 (Bray et al., 2015). Under the joint multidisciplinary diagnosis and treatment model of surgery, radiotherapy, chemotherapy, endocrinology, and targeted precision therapy, the mortality of advanced breast cancer or high-risk breast cancer with metastasis has not been significantly improved (Emens, 2018). All these data highlight the additional need for innovative methods to identify patients with high-risk disease.

Ferroptosis is a newly discovered form of regulating cell death, which is related to metabolism, redox biology, and human health. Emerging evidence suggests that it may trigger ferroptosis for cancer treatment, especially in eradicating aggressive malignancies that are resistant to traditional therapies (Liang et al., 2019). Previous studies have shown that ferroptotic cell death is a type of cell death that is different from apoptosis, various forms of necrosis, and autophagy. It is different in morphology, biochemistry, and genetics. Different from other forms of apoptosis and non-apoptotic death, the feature of this process is the iron-dependent accumulation of reactive oxygen species (ROS) (Yagoda et al., 2007; Christofferson and Yuan, 2010; Dixon et al., 2012). There have been some studies related to breast cancer and ferroptosis. Ma et al. discovered that ferroptosis was induced following siramesine and lapatinib treatment of breast cancer cells (Ma et al., 2016). Yu et al. found that target exosome-encapsulated erastin induced ferroptosis in triple-negative breast cancer cells (Yu et al., 2019b). There was also a study that explored that sulfasalazine-induced ferroptosis in breast cancer cells was reduced by the inhibitory effect of the estrogen receptor on the transferrin receptor (Yu et al., 2019a). In addition, Qiao et al. discovered that NR5A2 synergized with NCOA3 could induce breast cancer resistance to the BET inhibitor by regulating NRF2 to attenuate ferroptosis (Qiao et al., 2020). However, studies on the ferroptosis-related genes in the prognosis of breast cancer remain largely unknown.

In this study, we downloaded the mRNA expression data and corresponding clinical data of breast cancer patients from the TCGA database. Then, we constructed a prognostic ferroptosis-related gene signature based on the TCGA cohort and validated this model in the GEO cohort. We further did the functional enrichment analysis to identify the potential mechanisms.

## Materials and Methods

### Data Collection and Ferroptosis-Related Gene Definition

A total of 1057 breast cancer RNA expression data with clinical and follow-up information were downloaded from the TCGA cohort^1^. In addition, the breast cancer RNA expression data with clinical and follow-up information of three external validation cohorts were downloaded from the Gene Expression Omnibus (GEO) database^2^, including 327 samples of GSE20685, 88 samples of GSE20711, and 104 samples of GSE42568. The baseline clinical characteristics of the breast cancer patients in this study are shown in Table 1. All the data from TCGA and GEO were public. This study was exempted from the approval of the local ethics committees. The present study followed the TCGA and GEO data access policies and publication guidelines.

**TABLE 1:** The baseline clinical characteristics of the breast cancer patients in this study.

	**TCGA cohort**	**GEO cohort**			***P*-value**
		**GSE20685**	**GSE20711**	**GSE42568**	
No. of patients	1057	327	88	104	/
Age (median, range)	58 (26–90)	46 (24–84)	/	56 (31–90)	/
Stage (%)					
I	181	/	/	/	/
II	598	/	/	/	
III	237	/	/	/	
IV	30	/	/	/	
Unknown	11	/	/	/	
T					
1	278	101	/	/	0.092
2	607	188	/	/	
3	132	26	/	/	
4	37	12	/	/	
Unknown	3	0	/	/	
N					
0	497	137	/	/	<0.01
1	352	87	/	/	
2	119	63	/	/	
3	72	40	/	/	
Unknown	17	0	/	/	
M					
0	879	319	/	/	0.908
1	21	8	/	/	
Unknown	157	0	/	/	
Survival status					
OS days (median)	858	1862	2216	2197	/

*The P-value represents the comparison of clinicopathological parameters of patients between the TCGA cohort and GSE20685.*

Later, 259 ferroptosis-related genes were obtained from the FerrDb website^3^, which was the first database of ferroptosis regulators and markers and ferroptosis–disease associations. It classified ferroptosis-related genes into three subgroups, including drivers which were genes that promote ferroptosis, suppressors which were genes that prevent ferroptosis, and markers which were genes that indicated the occurrence of ferroptosis (Zhou and Bao, 2020). We further removed the duplicate genes of the three subgroups of ferroptosis gene sets and obtained a total of 259 genes for subsequent analysis. The detailed information and classification of ferroptosis-related genes are found in Supplementary Table 1.

### Construction and Validation of a Prognostic Ferroptosis-Related Gene Signature

The “limma” R package was used to identify the differentially expressed genes (DEGs) between breast cancer tissues and adjacent normal breast tissues; it was filtered by the cutoff values of false discovery rate (FDR) < 0.05 and log2| fold change| > 1 in the TCGA cohort. Later, we used univariate Cox analysis of overall survival (OS) to select the potential ferroptosis-related prognostic genes by R “survival” filtered by *p* < 0.05. In addition, the R “venn” package was used to get the intersect genes between ferroptosis-related DEGs and prognostic genes. We further used the intersect candidate genes for the construction of the prognostic signature. A ferroptosis-related prognostic signature-based prediction model was constructed by using coefficients (β) calculated from multivariate Cox regression as the weights. The risk score for each patient in the TCGA cohort was calculated based on the risk formula: risk score = expression of gene1 × β1 + expression of gene2 × β2 + … expression of genen × βn. The breast cancer samples were then divided into high-risk group and low-risk group according to the median value of risk scores. For the survival analysis, the optimal cutoff expression value was determined by the “survminer” R packages. We also used the “survivalROC” R package to conduct the time-dependent ROC curve analyses for evaluating the predictive power of our 11-gene signature prediction model. Besides, univariate and multivariate Cox regression analyses were used to explore whether the risk score calculated from our model could play as an independent prognostic factor for breast cancer patients after considering other clinical factors, including age, stage, T stage, N stage, and M stage.

### Functional Enrichment Analysis

The Gene Ontology (GO) and Kyoto Encyclopedia of Genes and Genomes (KEGG) were analyzed by the “clusterProfiler” R package through identifying the DEGs (| log2FC| ≥ 1, FDR < 0.05) between the high-risk and low-risk groups. Later, the ssGSEA algorithm was used to calculate the enrichment degree of 29 immune-related gene sets, including immune cell types, functions, or pathways. The annotated gene sets are concluded in Supplementary Table 2.

### Cell Culture

Human breast cancer cells MDA-MB-231 and MCF-7 were maintained in L15 and DMEM High Glucose (Gibco; Thermo Fisher Scientific, Inc., Waltham, MA, United States), respectively, and were supplied with 10% fetal bovine serum (FBS; Gibco; Thermo Fisher Scientific, Inc.) at 37°C with 5% CO_2_. The MCF-10A cells were cultured in DMEM/F12 (1:1) (Gibco; Thermo Fisher Scientific, Inc., Waltham, MA, United States) that was supplemented with 100 ng/ml cholera toxin (Sigma, St. Louis, MO, United States), 20 ng/ml epidermal growth factor (EGF) (Thermo Fisher Scientific, Inc., Waltham, MA, United States), 10 μg/ml insulin, 500 ng/ml hydrocortisone, and 5% heat-inactivated horse serum (all from Sigma) at 37°C with 5% CO_2_. BT-549 cells were cultured in 1640 medium (Gibco; Thermo Fisher Scientific, Inc., Waltham, MA, United States) with 10% FBS, 1μg/ml insulin (Sigma) at 37°C with 5% CO_2_. SUM-159 cells were maintained in DMEM/F12 with 10% FBS at 37°C with 5% CO_2_. The old cell culture medium was replaced with fresh medium every other day.

### Cell Viability Assay

MDA-MB-231 and MCF-7 cells were seeded in 96-well plates at a density of 3,000 cells per well and incubated in a humidified cell incubator at 37°C, 5% CO_2_ for 24 h. The cells were then treated with erastin (T-1765, TargetMol, Boston, MA, United States) of 0, 10, 20, and 40 μM for 24, 48, and 72 h. MCF-10A, BT-549, and SUM-159 cells were seeded in 96-well plates at a density of 3,000, 4,000, and 2,500 cells per well, respectively, and then incubated in a humidified cell incubator at 37°C, 5% CO_2_, for 24 h. The cells were then treated with erastin (T-1765, TargetMol) of 0, 10, 20, and 40 μM for 24 and 48 h. The Cell Counting Kit-8 (CCK-8) (TargetMol) reagent was then added, and the 96-well plate was continued to incubate for another 2 h. The OD (optical density) values were measured at 450 nm with a microplate reader. The experiments in all groups were performed in triplicates and repeated for three times.

### Detection of ROS

MDA-MB-231 and MCF-7 cells were incubated in a 60-mm dish for 24 h, then different treatment groups were treated with DMSO, a ferroptosis activator (erastin, 10, 20, 40 μM), for another 48 h. Later, cells were incubated in a 60-mm dish containing 5 μM BODIPY 581/591 C11 dye (D3861, Invitrogen, Carlsbad, CA, United States) for 30 min at 37°C; cells were washed with PBS and trypsinized, then stained with PI (propidium iodide) in PBS for 5 min. Cells were then subjected to flow cytometry analysis using flow cytometry. The FL1 channel signal in live cells was plotted as shown in the figures.

### RNA Isolation and Real-Time PCR

Total RNA from MDA-MB-231, MCF-7, MCF-10A, BT-549, and SUM-159 cells treated with erastin was extracted with the RNeasy Mini Kit (Qiagen, Valencia, CA, United States) according to the manufacturer’s protocol. The concentration and purity of all RNA samples were determined at an absorbance ratio of 260/280 nm. A total of 1 μg RNA was reverse-transcribed using iScript cDNA Synthesis kit from Bio-Rad^TM^ (Hercules, CA, United States). Real-time PCR analysis was set up with the SYBR Green qPCR Supermix kit (Invitrogen, Carlsbad, CA, United States) and carried out in the iCycler thermal cycler. It was used to detect the mRNA expression levels of core prognostic genes. The relative level of mRNA expression of a gene was determined by normalizing with GAPDH. The primers used for real-time PCR are listed in Supplementary Table 3 and were purchased from Sangon Biotech (Shanghai, China).

### Western Blot

The proteins from MDA-MB-231, MCF-7, MCF-10A, BT-549, and SUM-159 cells treated with erastin were lysed in RIPA buffer, containing the phosphatase and protease inhibitor, by using an ultrasonic crusher (Sonics & Materials, Inc., Newtown, CT, United States). Later, the cell mixture was kept on ice for 30 min and transferred to a centrifuge tube for centrifugation at 4°C, 12,000 rpm, for 20 min. The breast cancer proteins from the upper part of the supernatant were collected and detected by BCA Protein Assay Kit (Pierce; Thermo Fisher Scientific, Inc.). Thirty-five micrograms of protein was separated on 10% SDS-PAGE gels and transferred to polyvinylidene fluoride (PVDF) membranes. Then, the PVDF membranes were blocked with 5% non-fat milk in TBST to prevent non-specific binding and subsequently incubated with a primary antibody (G6PD: cat no. sc-373886; Santa Cruz Biotechnology, Inc., Dallas, TX, United States; dilution, 1:500; BRD4: cat no. sc-518021; Santa Cruz Biotechnology, Inc.; dilution, 1:500; PI 3-kinase p110: cat no. sc-8010; Santa Cruz Biotechnology, Inc.; dilution, 1:500; GAPDH: cat no. 5174; Cell Signaling Technology, Inc., Danvers, MA, United States; dilution, 1:1,000) overnight at 4°C. All samples were incubated with anti-horseradish peroxidase-linked IgG secondary antibody (cat no. 7074; Cell Signaling Technology, Inc.; dilution, 1:2,000) at room temperature for 2 h and detected *via* chemiluminescence detection system (version 3.0; Bio-Rad Laboratories, Inc., Hercules, CA, United States).

### Plasmids and Cell Transfection

pcDNA3.1 G6PD plasmid was kindly provided by Professor Ke Wang (XJTU). To overexpress G6PD, MDA-MB-231 cells were transfected with the empty vector pcDNA3.1 and pcDNA3.1 encoding G6PD, and cells were yielded to Western blot for transfect efficiency verification and then subjected to the following experiment. Transfections were performed using Lipofectamine 3000 (Invitrogen) according to the manufacturer’s instructions.

### Statistical Analysis

All data were analyzed using R version 4.0.2 or GraphPad Prism 8, and all experiments were repeated at least three times. These results were presented as mean ± standard deviation (SD). We used Student’s *t*-test to compare the gene expression between tumor tissues and adjacent normal tissues. The Mann–Whitney test with *P*-values was used to compare the ssGSEA scores of immune cells and pathways between the high-risk and low-risk groups. Kaplan–Meier analysis with the log-rank test was used to compare the OS between the high-risk and low-risk groups. Besides, univariate and multivariate Cox regression analyses were used to identify the independent predictors of OS. The comparison of patients’ clinicopathological parameters between the TCGA cohort and GSE20685 was analyzed by the χ^2^-test. All statistical analyses were performed with R software (Version 4.0.2). All *P* < 0.05 was considered statistically significant.

## Results

**FIGURE 1:**
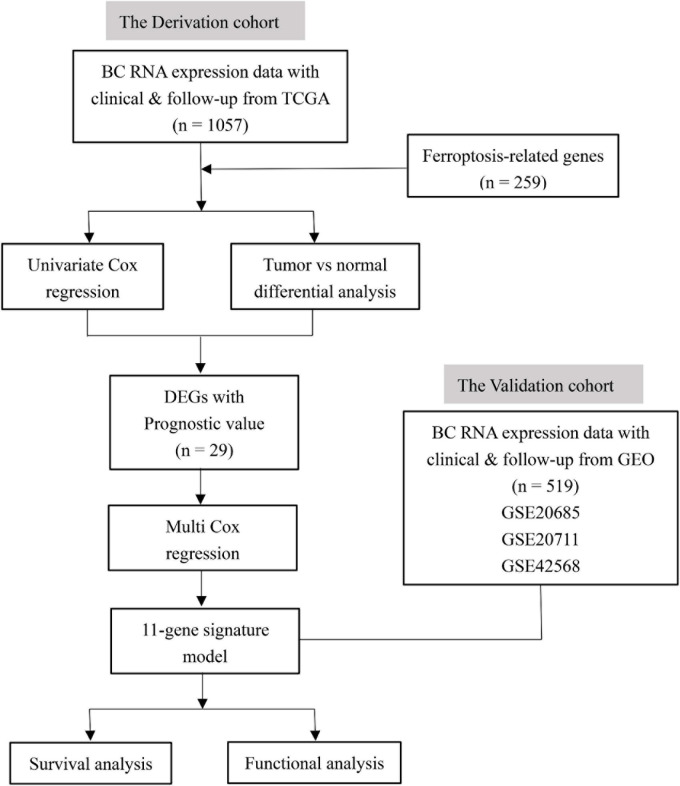
The flowchart of construction and validation of data collection and analysis.

Figure 1 shows the flowchart of construction and validation of data collection and analysis. We totally enrolled 1,057 breast cancer patients from the TCGA as the derivation cohort and 519 breast cancer patients from GEO as the validation cohort. The baseline clinical characteristics of the breast cancer patients in this study are summarized in Table 1.

### Identification of Prognostic Ferroptosis-Related DEGs in the TCGA Cohort

**FIGURE 2:**
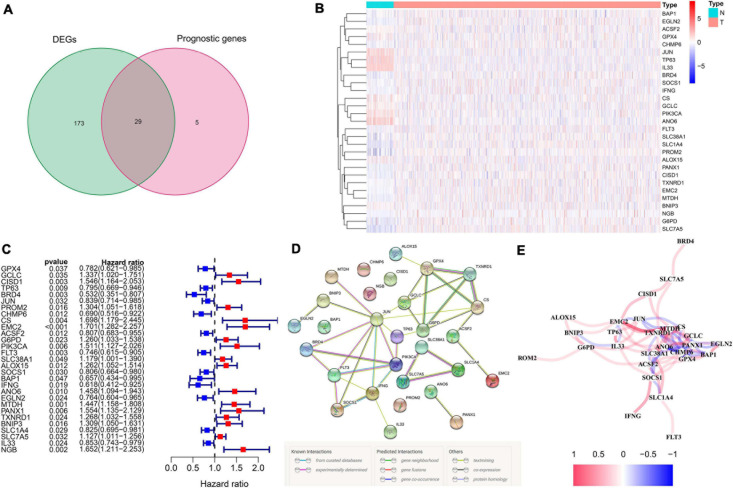
The identification of the candidate ferroptosis-related genes in the TCGA cohort. **(A)** Differentially expressed genes between breast cancer and adjacent normal breast tissue that were correlated with OS are shown by a Venn diagram. **(B)** The heatmap of the 29 overlapping genes’ expression in breast cancer tissues. **(C)** The univariate Cox regression analysis between gene expression and OS are shown by the forest plots. **(D)** The interactions among candidate genes are shown by the PPI network through the STRING database. **(E)** The correlation network of selected candidate genes (different colors represented different degrees of correlation coefficient).

We totally included 259 well-defined ferroptosis-related genes in this study, including 108 drivers that promote ferroptosis, 69 suppressors that prevent ferroptosis, and 111 markers that indicate the occurrence of ferroptosis. Supplementary Table 1 shows the detailed information of ferroptosis-related gene sets that were used in our analyses. After the identification of prognostic ferroptosis-related DEGs in the TCGA cohort, results showed that most of the ferroptosis-related genes (202/259, 78%) were differentially expressed between breast cancer tumor tissues and adjacent non-tumorous tissues, including 29 of them which were significantly correlated with OS in the univariate Cox regression analysis (Figure 2A). A total of 29 prognostic ferroptosis-related DEGs were identified by the criteria of FDR < 0.05 (Figures 2B,C). The heatmap in Figure 2B depicts the expression level and distribution of those 29 prognostic ferroptosis-related DEGs. The forest plot of Figure 2C shows the results of univariate Cox regression analysis of these 29 genes. The results demonstrated that 13 of these genes played protective roles in breast cancer patients with HR less than 1 (GPX4, TP63, BRD4, JUN, CHMP6, ACSF2, FLT3, SOCS1, BAP1, IFNG, EGLN2, SLC1A4, IL33), while the other 16 genes (GCLC, CISD1, PROM2, CS, EMC2, G6PD, PIK3CA, SLC38A1, ALOX15, ANO6, MTDH, PANX1, TXNRD1, BNIP3, SLC7A5, NGB) were risk factors with HR more than 1. We further used the STRING database to detect the protein–protein interaction network among those 29 genes (Figure 2D). The interaction network indicated that JUN, FLT3, PIK3CA, G6PD, and GPX4 were the hub genes. The correlation network of the selected candidate genes is shown in Figure 2E, among which different colors represented different degrees of the correlation coefficient.

### Construction of the Ferroptosis-Related Prognostic Signature in TCGA Cohort

**FIGURE 3:**
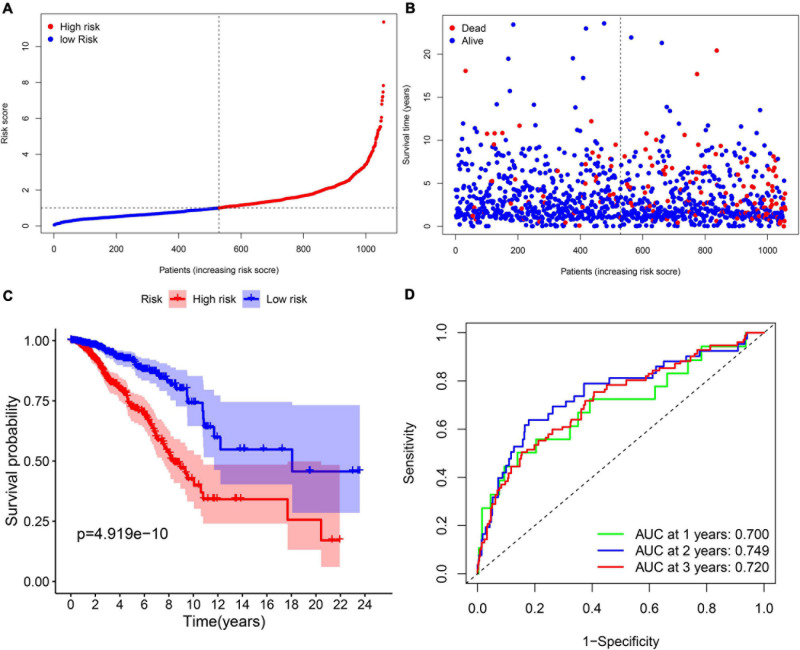
The prognostic analysis of the 11-gene signature model in the TCGA cohort. **(A)** The distribution and median value of the risk scores in the TCGA cohort. **(B)** The distribution of OS, OS status, and risk score in the TCGA cohort. **(C)** The Kaplan–Meier curves for the OS of patients in the TCGA cohort which was divided into high-risk group and low-risk group. **(D)** The AUC of time-dependent ROC curves was used to verify the prognostic performance of the risk score in the TCGA cohort.

Multi-Cox regression analysis was applied to construct an 11-gene prognostic model using the expression of 29 genes mentioned above. The risk score could be calculated by the following formula: risk score = 0.366 ^∗^ expression level of CISD1 + (–0.272) ^∗^ expression level of TP63 + (–0.499) ^∗^ expression level of BRD4 + 0.290 ^∗^ expression level of PROM2 + 0.277 ^∗^ expression level of EMC2 + 0.217 ^∗^ expression level of G6PD + 0.294 ^∗^ expression level of PIK3CA + (–0.179) ^∗^ expression level of FLT3 + (–0.666) ^∗^ expression level of IFNG + 0.500 ^∗^ expression level of ANO6 + (–0.275) ^∗^ expression level of SLC1A4. We stratified all breast cancer patients in the TCGA cohort into the high-risk group (*n* = 528) and low-risk (*n* = 529) group according to the median value of risk scores (Figure 3A). In addition, Figure 3B reflects that patients with high-risk scores were more likely to die earlier than those with-low risk scores. Consistently, the Kaplan–Meier survival curve in Figure 3C shows that OS of breast cancer patients in the high-risk group was significantly worse than those in the low-risk group (*p* = 4.919e–10). We further evaluated the predictive efficacy of the prognostic signature for OS in breast cancer patients by the time-dependent ROC curves, and the area under the curve (AUC) reached 0.700 at 1 year, 0.749 at 2 years and 0.720 at 3 years (Figure 3D).

### Validation of the Ferroptosis-Related Prognostic Signature in GEO Cohort

**FIGURE 4:**
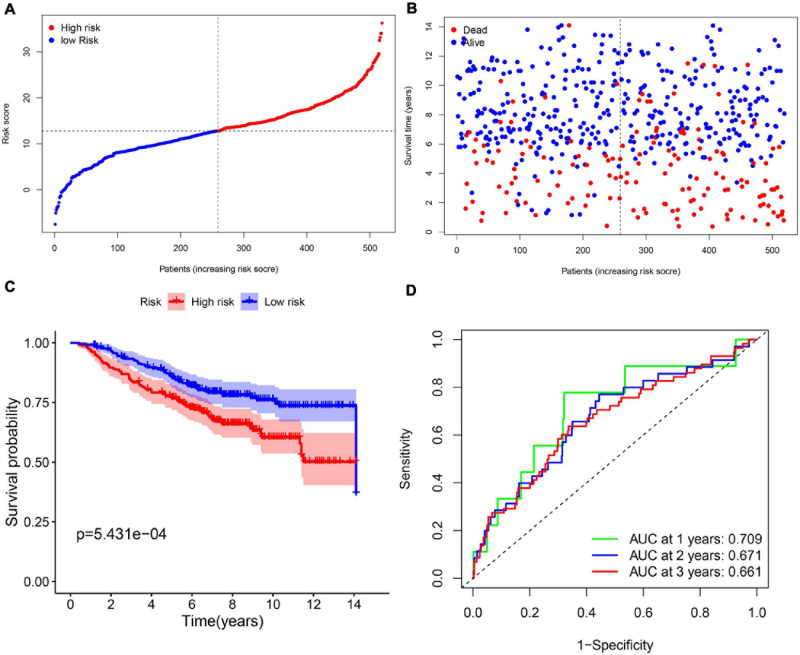
The validation of the 11-gene signature model in the GEO cohort. **(A)** The distribution and median value of the risk scores in the GEO cohort. **(B)** The distribution of OS, OS status, and risk score in the GEO cohort. **(C)** The Kaplan–Meier curves for the OS of patients in the GEO cohort which was divided into high-risk group and low-risk group. **(D)** The AUC of time-dependent ROC curves in the GEO cohort.

In order to test the robustness of the 11-gene model constructed from the TCGA cohort, GEO cohorts (GSE20685, GSE20711, and GSE42568) from the GEO database were used for external validation. The patients from the GEO cohort were divided into the high-risk group and low-risk group by the median value of risk scores calculated with the same formula from the TCGA cohort (Figure 4A). Similar to the results that were obtained from the TCGA cohort, in the GEO validation cohort, patients with high-risk scores had a higher probability of death earlier than the low-risk group (Figure 4B). Likewise, the Kaplan–Meier survival curve showed that OS of breast cancer patients in the high-risk group was worse than that in the low-risk group with statistical significance (*p* = 5.431e-04). In addition, the AUC of the 11-gene signature was 0.709 at 1 year, 0.671 at 2 years, and 0.661 at 3 years (Figures 4C,D).

### Independent Prognostic Value of the 11-Gene Ferroptosis-Related Prognostic Signature

**FIGURE 5:**
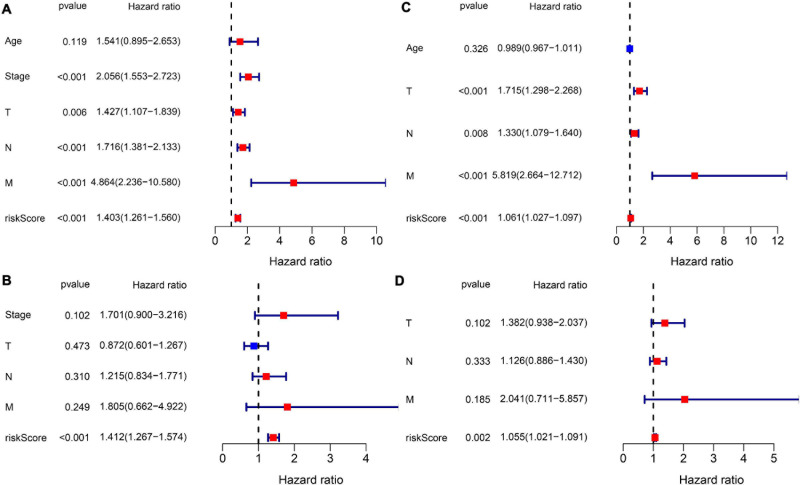
Results of the Cox regression analyses regarding OS in the TCGA derivation cohort and GEO validation cohort. **(A)** The univariate Cox regression analyses regarding OS in the TCGA derivation cohort. **(B)** The multivariate Cox analyses regarding OS in the TCGA derivation cohort. **(C)** The univariate Cox regression analyses regarding OS in the GEO validation cohort. **(D)** The multivariate Cox analyses regarding OS in the GEO validation cohort.

Subsequently, we extracted the available clinical variables in the TCGA database and GEO database. Univariate and multivariate Cox regression analyses were used to detect whether the risk score could play an independent prognostic role in predicting OS of breast cancer. Figures 5A,C show that the risk score was an independent prognostic predictor for OS in both TCGA cohort (HR = 1.403, 95% CI = 1.261–1.560, *P* < 0.001) and GEO cohort (GSE20685: HR = 1.061, 95% CI = 1.027–1.097, *P* < 0.001). Multivariate Cox regression further proved that our risk score could work as an independent predictor for OS in both cohorts (TCGA: HR = 1.412, 95% CI = 1.267–1.574, *P* < 0.001; GEO: HR = 1.055, 95% CI = 1.021–1.091, *P* = 0.002) (Figures 5B,D).

### Functional Analyses of the Prognostic Signature-Related Biological Pathways

**FIGURE 6:**
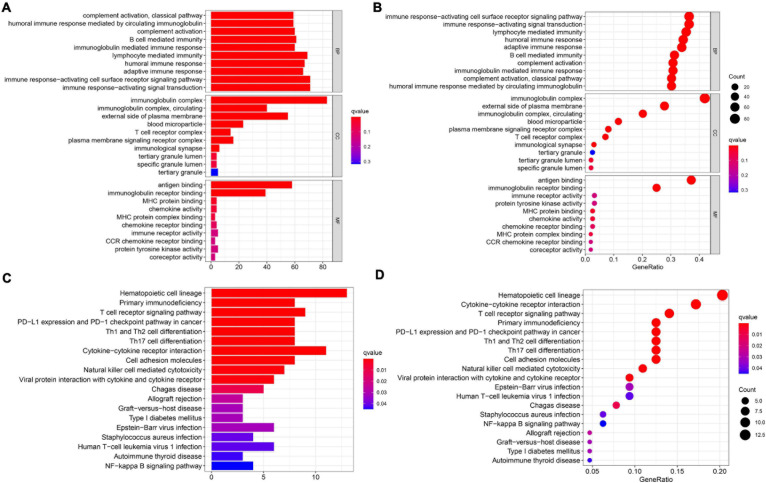
Results of GO and KEGG analyses in the TCGA cohort. **(A,B)** The significant GO enrichment in the TCGA cohort. **(C,D)** The significant KEGG pathways in the TCGA cohort.

In order to elucidate the underlying biological functions and pathways that were correlated with our 11-gene prognostic signature-related model, we performed GO enrichment and KEGG pathway analyses by analyzing the DEGs between the high-risk and low-risk groups. Interestingly, results showed that the DEGs from the TCGA cohort were obviously enriched in many immune-related biological processes in GO enrichment (Figures 6A,B). The representing immune-related biological processes and molecular functions include immunoglobulin-mediated immune response, lymphocyte-mediated immunity, humoral immune response, adaptive immune response, immune response-activating cell surface receptor signaling pathway, and immune response-activating signal transduction. Similarly, the KEGG pathway analyses also indicated immune-related pathways such as primary immunodeficiency, T cell receptor signaling pathway, PD-L1 expression and PD-1 checkpoint pathway in cancer, Th1 and Th2 cell differentiation, and Th17 cell differentiation (Figures 6C,D).

**FIGURE 7:**
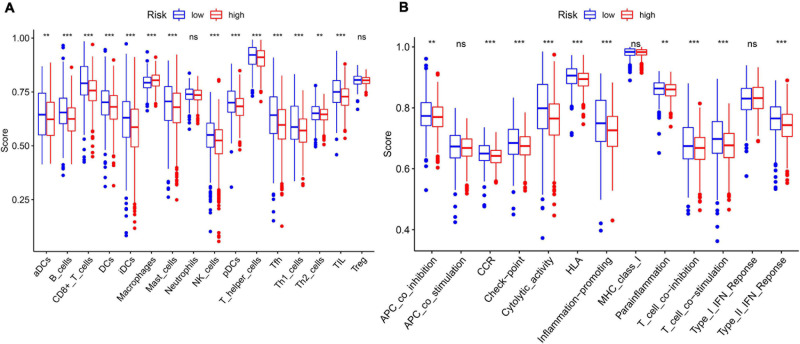
The ssGSEA scores between different risk groups in the TCGA cohort. **(A)** The scores of 16 immune cells in the boxplot. **(B)** The 13 immune-related functions in boxplots. The adjusted *P*-values are shown in the analyses. ns, not significant; **P < 0.01; ***P < 0.001.

In order to further explore the correlation between the 11-gene signature risk score and immune status, we used ssGSEA to quantify the enrichment scores of different immune cell subgroups, related functions, or pathways. As we expected, the contents of the antigen presentation process showed statistical difference between the high-risk group and low-risk group in the TCGA cohort, including aDCs, B cells, CD8 + T cells, DCs, iDCs, macrophages, mast cells, NK cells, pDCs, T helper cells, Tfh, Th1 cells, Th2 cells, TIL, APC co-inhibition, cytokine–cytokine receptor (CCR), checkpoint, cytolytic activity, HLA, T cell co-inhibition, and T cell co-stimulation (Figure 7). In particular, the scores of Th1 cells, Th2 cells, and T cell co-inhibition and co-stimulation were reflected significantly different between the high-risk group and low-risk group; this finding could correspond with the above enrichment in KEGG analysis.

### Effects of Erastin on the Expression Levels of 11 Core Prognostic Genes in Breast Cancer Cell Lines *in vitro*

**FIGURE 8:**
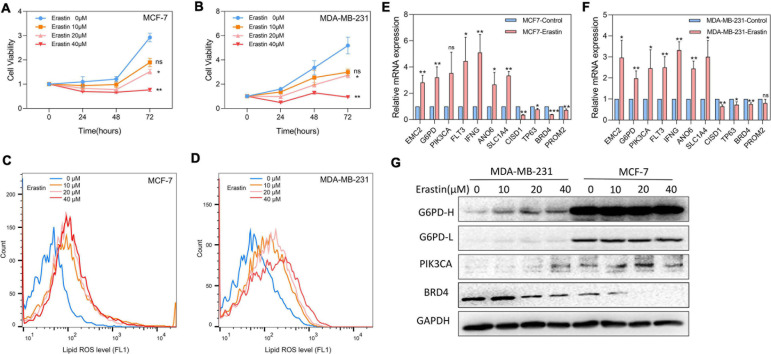
Effects of ferroptosis inducer on expression levels of 11-core prognostic genes in breast cancer cell lines *in vitro*. **(A,B)** The cell viability of MCF-7 **(A)** and MDA-MB-231 **(B)** treated by erastin was tested by CCK8 assay. **(C,D)** Lipid peroxidation of MCF-7 **(C)** and MDA-MB-231 **(D)** treated by erastin was assessed by flow cytometry after C11-BODIPY staining. **(E,F)** The expression changes of 11-core prognostic genes in MCF-7 **(E)** and MDA-MB-231 **(F)** after being treated with 20 μM erastin were detected by real time-PCR. **(G)** The expression changes of G6PD, PIK3CA, and BRD4 genes in MDA-MB-231 and MCF-7 after being treated with erastin for 48 h were detected by western blot (G6PD-H means high exposure, G6PD-L means short exposure). * *P* < 0.05; **P < 0.01; ***P < 0.001.

Breast cancer cell lines MDA-MB-231 and MCF-7 were then treated with ferroptosis inducer erastin in order to explore the role of the 11-core prognostic genes. In Figure 8A,B, the cell viability assay showed that the ferroptosis inducer erastin could inhibit the proliferation of MDA-MB-231 and MCF-7 in a dose-dependent manner. The experiment results showed that the growth inhibitory effects of erastin in both breast cancer cell lines were statistically significant when the concentration was over 20 μM. The growth of MDA-MB-231 and MCF-7 was almost completely inhibited when the erastin treatment concentration reached 40 μM. The correspondence images of MCF-7’s cell viability and MDA-MB-231 treated by erastin are found in Supplementary Figure 1. In addition, more cell lines such as MCF-10A, BT-549, and SUM-159 were used to test erastin’s inhibition on cell viability. Similar results are found in Supplementary Figure 2. Later, we detected that erastin treatment at 10, 20, and 40 μM could significantly increase ROS accumulation by FACS (Figures 8C,D). Finally, RT-PCR was used to investigate the expression of 11-core prognostic genes in MDA-MB-231 and MCF-7 after being treated with erastin of 20 μM for 48 h. Results showed that mRNA expression levels of six genes (EMC2, G6PD, FLT3, IFNG, ANO6, and SLC1A4) were increased and three genes (CISD1, TP63, and BRD4) were decreased after erastin treatment. However, the increase of PIK3CA was not statistically significant in MCF-7-erastin cells and PROM2 was barely decreased in MDA-MB-231 cells (Figures 8E,F). The results indicated that EMC2, G6PD, FLT3, IFNG, ANO6, and SLC1A4 were positively correlated with ferroptosis while CISD1, TP63, and BRD4 were negatively correlated with ferroptosis in breast cancer. Western blotting of G6PD, PIK3CA, and BRD4 was performed to test future PCR results (Figure 8G). Our results showed that the protein alteration was consistent with the PCR result except G6PD in MCF-7, which indicated that our 11-gene model could be verified by cell line experiment. Since G6PD produces NADPH which is a scavenger of ROS, we also overexpressed G6PD in MDA-MB-231 to detect erastin sensitivity. The results suggested that overexpression of G6PD would result in decrease in the sensitivity of cells to erastin (Supplementary Figure 3). In addition, more cell lines such as MCF10A, BT549, and SUM159 were used to detect our 11-gene alteration after adding erastin; similar results are found in Supplementary Figure 4. This result indicated the different stages of breast cancer cell lines with ferroptotic inducers had the same trend.

## Discussion

In our study, we explored a total of 259 well-defined ferroptosis-related genes in breast cancer tissues and their correlations with OS. A novel 11-gene ferroptosis-related prognostic model was constructed and validated by the external cohort. We further did functional analyses and surprisingly found some immune-related biological processes.

A few studies have discovered that certain drugs might cause ferroptosis in breast cancer (Ma et al., 2016; Yu et al., 2019a, b); however, there are still few studies on whether there exist specific ferroptosis-related genes in regulating the progression of breast cancer. The relationship between ferroptosis and the prognosis of breast cancer remains to be clarified. Interestingly, 78% ferroptosis-related genes were differentially expressed between breast cancer tumor tissue and adjacent non-tumorous tissues, including 29 of them which were significantly correlated with OS in the univariate Cox regression analysis. These results fully demonstrated that ferroptosis might play an important role in breast cancer and the possibility of using these ferroptosis-related genes to establish a prognostic model.

Our 11-gene prognostic signature-related model includes CISD1, TP63, BRD4, PROM2, EMC2, G6PD, PIK3CA, FLT3, IFNG, ANO6, and SLC1A4. These genes could be classified into three subgroups, including drivers (EMC2, G6PD, PIK3CA, FLT3, IFNG, ANO6) which were genes that promote ferroptosis, suppressors (CISD1, TP63, BRD4, PROM2) which were genes that prevent ferroptosis, and markers (SLC1A4) which were genes that indicated the occurrence of ferroptosis. By using an shRNA library that targets plenty of genes which encode mitochondrial proteins, EMC2 (ER membrane protein complex subunit 2, also referred as TTC35) has been identified to play an important role in erastin-induced ferroptosis in HT-1080 fibrosarcoma cells (Dixon et al., 2012). G6PD (glucose-6-phosphate dehydrogenase) is involved in the pentose phosphate pathway of energy metabolism and has been verified to prevent erastin-induced ferroptosis in human lung adenocarcinoma cells while it was knocked down by the corresponding shRNA (Dixon et al., 2012). Our result also showed that overexpression of G6PD can reduce sensitivity to erastin in MDA-MB-231. We considered the increase in G6PD as a compensatory adjustment to erastin-induced ferroptosis which is consistent with their study. Kang et al. demonstrated that both FLT3 and PIK3CA inhibitors play protective roles against glutamate-induced oxidative stress and prove the involvement of lipid peroxidation-mediated ferroptosis (Kang et al., 2014). In addition, IFNG releases from CD8 + T cells could downregulate two subunits of glutamate-cystine antiporter system xc-, restrain tumor cell cystine uptake, and finally promote tumor cell lipid peroxidation and ferroptosis (Wang et al., 2019). German scientists discovered that ANO6 is activated during erastin and RSL3-induced ferroptosis; however, inhibition of ANO6 could also lead to cell death. They concluded that the activation of ANO6 might be an important component during ferroptosis cell death and induce cell death in cancer cells (Ousingsawat et al., 2019). In contrast, CISD1, TP63, BRD4, and PROM2 were all genes that prevent ferroptosis. It has been explored that genetic inhibition of CISD1 (CDGSH iron sulfur domain 1, also termed mitoNEET) could increase iron-mediated intramitochondrial lipid peroxidation, resulting in erastin-induced ferroptosis. On the contrary, pioglitazone stabilizes the iron sulfur cluster of CISD1 and inhibits the mitochondrial iron uptake as well as lipid peroxidation which finally leads to ferroptosis (Yuan et al., 2016). TP63 has been validated to inhibit oxidative stress-induced cell death ferroptosis through cooperating with the BCL-2 family to promote clonogenic survival (Wang et al., 2017). In addition, after knockdown of BRD4 or under (+)-JQ1 (an inhibitor of the tumor-driver bromodomain protein BRD4), the expressions of ferroptosis-associated genes GPX4, SLC7A11, and SLC3A2 are downregulated. It indicated that knockdown or inhibition of BRD4 would induce ferroptosis *via* ferritinophagy or regulating ferroptosis-associated genes through epigenetic repression of BRD4 (Sui et al., 2019). Furthermore, Brown explored that PROM2 could facilitate ferroptosis resistance in mammary epithelial and breast cancer cell lines; in detail, PROM2 promotes the formation of ferritin-containing multivesicular bodies and exosomes which transport iron out of the cell, therefore, inhibiting ferroptosis (Brown et al., 2019). Besides, SLC1A4 was also indicated to correlate with the occurrence of ferroptosis (Dixon et al., 2014). These genes are all associated with the promotion or prevention of ferroptosis in different cancers through multiple mechanisms; however, whether these genes play a vital role in the prognosis of breast cancer patients through influencing ferroptosis remains unclear.

There are already some researches surrounding the ferroptosis and cancer progression mechanisms in the recent years; however, the potential relationship between ferroptosis and cancer immunity remains to be elucidated. In our study, we performed GO enrichment and KEGG pathway analyses by analyzing the DEGs between the high-risk and low-risk groups based on our 11-gene model. Interestingly, we discovered some immune-related biological processes in GO enrichment. These gene enrichment analyses suggested that our ferroptosis might be closely related to the immune response of breast cancer. Besides, our results also revealed that breast cancer patients in the high-risk group had decreased infiltration degrees of B cells, CD8^+^ T cells, dendritic cells, T helper cells, Tfh cells, Th1 cells, Th2 cells, and TIL. The risk score formed by our 11-gene signature is negatively correlated with immune cell infiltration, suggesting that this model might be a predictor of local immune response in the tumor bed. At present, studies have shown that tumor-infiltrating B cells express and secrete antibodies which could promote tumor cell lysis and apoptosis (Ruffell et al., 2012). The direct and indirect cytotoxic effects of CD8^+^ T cells support the findings that CD8 + T cell infiltration is associated with good survival outcomes (Mahmoud et al., 2011; Matsumoto et al., 2016). There has been a discovery that neutrophil infiltration is also an independent prognostic factor which correlated with better overall survival in breast cancer (Zeindler et al., 2019). However, the infiltration level of neutrophil in the low-risk group and high-risk group of our model states no statistical significance. It is well known that dendritic cell-mediated T cell activation is a key step in antitumor immunity. Studies have found that the functional defects of dendritic cells in patients with early breast cancer might be an important factor in tumor progression (Satthaporn et al., 2004). Consistent with our research, in the ferroptosis-related model we constructed, the proportion of dendritic cells in breast cancer patients in the high-risk group was significantly reduced in the low-risk group. In summary, the imbalance of immune infiltration and immune response dysfunction in the surrounding environment of the tumor may be at least partially attributed to the high-risk score.

Besides, there also exist some limitations in our study. Firstly, our ferroptosis-related prognostic model was constructed and validated in public databases with retrospective data. We will further use our prospective multicenter clinical data for further verification in the future. Secondly, we only used ferroptosis-related genes to construct this prognostic model; therefore, many other hot biomarkers might be excluded. Also, the correlation between our model and immune-related pathways needed further experimental validation.

## Conclusion

In conclusion, a novel ferroptosis-related gene model can be used for prognostic prediction in breast cancer. New ferroptosis-related genes might be used for breast cancer targeting therapy in the future.

## Data Availability Statement

The datasets supporting the results and conclusions of this study were downloaded from the TCGA (http://tcga-data.nci.nih.gov/tcga/) and GEO (http://www.ncbi.nlm.nih.gov/geo/), and we thank The Cancer Genome Atlas (TCGA) and Gene Expression Omnibus (GEO) for providing transcriptomics and clinicopathological data.

## Author Contributions

LZ: collection and assembly of data, data analysis andinterpretation, manuscript writing, and methodology and software. QT: collection and assembly of data, data analysis and interpretation, and manuscript writing and editing. SJ and HG: data analysis, manuscript writing and interpretation. SY and YZ: manuscript writing and project administration. YY: manuscript writing. YR: conception and design. JH and BW: conception and design, supervision and editing. All authors: article writing and final approval of article.

## Conflict of Interest

The authors declare that the research was conducted in the absence of any commercial or financial relationships that could be construed as a potential conflict of interest.

## Footnotes

1 https://portal.gdc.cancer.gov

2 http://www.ncbi.nlm.nih.gov/geo

3 http://www.zhounan.org/ferrdb

## Supplementary Material

Supplementary Figure 1 The images about MCF7 cell viability and MDA-MB-231 treated by erastin.

Supplementary Figure 2 The cell viability of MCF-10A **(A)**, BT-549 **(B)**, and SUM-159 **(C)** treated by erastin was tested by CCK8 assay.

Supplementary Figure 3 The transfection of overexpression plasmid G6PD in MDA-MB-231 and the cell viability test. **(A)** The transfection efficiency was tested by western blot. **(B)** The viability of MDA-MB-231 con and MDA-MB-231 overexpression G6PD were tested by CCK8 assay.

Supplementary Figure 4 Effects of ferroptosis inducer on expression levels of 11 core prognostic genes in breast cancer cell lines *in vitro*. **(A–C)** The expression changes of 11 core prognostic genes in MCF-10A **(A)**, BT-549 **(B)**, and SUM-159 **(C)** after treated with 20μM erastin were detected by real time-PCR. **(D)** The expression changes of G6PD, PIK3CA, and BRD4 genes in MCF-10A, BT-549, and SUM-159 after treated with erastin for 48 h was detected by western blot.

## References

[B1] Bray F. Ferlay J. Laversanne M. Brewster D. H. Gombe Mbalawa C. Kohler B. (2015). Cancer incidence in five continents: inclusion criteria, highlights from volume X and the global status of cancer registration. *Int. J. Cancer* 137 2060–2071. 10.1002/ijc.29670 26135522

[B2] Brown C. W. Amante J. J. Chhoy P. Elaimy A. L. Liu H. Zhu L. J. (2019). Prominin2 drives ferroptosis resistance by stimulating iron export. *Dev. Cell* 51 575–586. 10.1016/j.devcel.2019.10.007 31735663 PMC8316835

[B3] Christofferson D. E. Yuan J. (2010). Necroptosis as an alternative form of programmed cell death. *Curr. Opin. Cell Biol.* 22 263–268. 10.1016/j.ceb.2009.12.003 20045303 PMC2854308

[B4] Dixon S. J. Lemberg K. M. Lamprecht M. R. Skouta R. Zaitsev E. M. Gleason C. E. (2012). Ferroptosis: an iron-dependent form of nonapoptotic cell death. *Cell* 149 1060–1072. 10.1016/j.cell.2012.03.042 22632970 PMC3367386

[B5] Dixon S. J. Patel D. N. Welsch M. Skouta R. Lee E. D. Hayano M. (2014). Pharmacological inhibition of cystine-glutamate exchange induces endoplasmic reticulum stress and ferroptosis. *Elife* 3:e02523. 10.7554/eLife.02523 24844246 PMC4054777

[B6] Emens L. A. (2018). Breast cancer immunotherapy: facts and hopes. *Clin. Cancer. Res.* 24 511–520. 10.1158/1078-0432.CCR-16-3001 28801472 PMC5796849

[B7] Harbeck N. Penault-Llorca F. Cortes J. Gnant M. Houssami N. Poortmans P. (2019). Breast cancer. *Nat. Rev. Dis. Primers* 5:66. 10.1038/s41572-019-0111-2 31548545

[B8] Kang Y. Tiziani S. Park G. Kaul M. Paternostro G. (2014). Cellular protection using Flt3 and PI3Kα inhibitors demonstrates multiple mechanisms of oxidative glutamate toxicity. *Nat. Commun.* 5:3672. 10.1038/ncomms4672 24739485 PMC4128233

[B9] Liang C. Zhang X. Yang M. Dong X. (2019). Recent progress in ferroptosis inducers for cancer therapy. *Adv. Mater.* 31:e1904197. 10.1002/adma.201904197 31595562

[B10] Ma S. Henson E. S. Chen Y. Gibson S. B. (2016). Ferroptosis is induced following siramesine and lapatinib treatment of breast cancer cells. *Cell Death Dis.* 7:e2307. 10.1038/cddis.2016.208 27441659 PMC4973350

[B11] Mahmoud S. M. Paish E. C. Powe D. G. Macmillan R. D. Grainge M. J. Lee A. H. (2011). Tumor-infiltrating CD8+ lymphocytes predict clinical outcome in breast cancer. *J. Clin. Oncol.* 29 1949–1955. 10.1200/JCO.2010.30.5037 21483002

[B12] Matsumoto H. Thike A. A. Li H. Yeong J. Koo S. L. Dent R. A. (2016). Increased CD4 and CD8-positive T cell infiltrate signifies good prognosis in a subset of triple-negative breast cancer. *Breast Cancer Res. Treat.* 156 237–247. 10.1007/s10549-016-3743-x 26960711

[B13] Ousingsawat J. Schreiber R. Kunzelmann K. (2019). TMEM16F/Anoctamin 6 in ferroptotic cell death. *Cancer* 11:625. 10.3390/cancers11050625 31060306 PMC6562394

[B14] Qiao J. Chen Y. Mi Y. Jin H. Huang T. Liu L. (2020). NR5A2 synergizes with NCOA3 to induce breast cancer resistance to BET inhibitor by upregulating NRF2 to attenuate ferroptosis. *Biochem. Biophys. Res. Commun.* 530 402–409. 10.1016/j.bbrc.2020.05.069 32536370

[B15] Ruffell B. Au A. Rugo H. S. Esserman L. J. Hwang E. S. Coussens L. M. (2012). Leukocyte composition of human breast cancer. *Proc. Natl. Acad. Sci. U.S.A.* 109 2796–2801. 10.1073/pnas.1104303108 21825174 PMC3287000

[B16] Satthaporn S. Robins A. Vassanasiri W. El-Sheemy M. Jibril J. A. Clark D. (2004). Dendritic cells are dysfunctional in patients with operable breast cancer. *Cancer Immunol. Immunother.* 53 510–518. 10.1007/s00262-003-0485-5 14740176 PMC11032822

[B17] Sui S. Zhang J. Xu S. Wang Q. Wang P. Pang D. (2019). Ferritinophagy is required for the induction of ferroptosis by the bromodomain protein BRD4 inhibitor (+)-JQ1 in cancer cells. *Cell Death Dis.* 10:331. 10.1038/s41419-019-1564-7 30988278 PMC6465411

[B18] Wang G. X. Tu H. C. Dong Y. Skanderup A. J. Wang Y. Takeda S. (2017). ΔNp63 Inhibits oxidative stress-induced cell death, including ferroptosis, and cooperates with the BCL-2 family to promote clonogenic survival. *Cell Rep.* 21 2926–2939. 10.1016/j.celrep.2017.11.030 29212036 PMC5915869

[B19] Wang W. Green M. Choi J. E. Gijón M. Kennedy P. D. Johnson J. K. (2019). CD8+ T cells regulate tumour ferroptosis during cancer immunotherapy. *Nature* 569 270–274. 10.1038/s41586-019-1170-y 31043744 PMC6533917

[B20] Yagoda N. von Rechenberg M. Zaganjor E. Bauer A. J. Yang W. S. Fridman D. J. (2007). RAS-RAF-MEK-dependent oxidative cell death involving voltage-dependent anion channels. *Nature* 447 864–868. 10.1038/nature05859 17568748 PMC3047570

[B21] Yu H. Yang C. Jian L. Guo S. Chen R. Li K. (2019a). Sulfasalazine-induced ferroptosis in breast cancer cells is reduced by the inhibitory effect of estrogen receptor on the transferrin receptor. *Oncol. Rep.* 42 826–838. 10.3892/or.2019.7189 31173262

[B22] Yu M. Gai C. Li Z. Ding D. Zheng J. Zhang W. (2019b). Targeted exosome-encapsulated erastin induced ferroptosis in triple negative breast cancer cells. *Cancer Sci.* 110 3173–3182. 10.1111/cas.14181 31464035 PMC6778638

[B23] Yuan H. Li X. Zhang X. Kang R. Tang D. (2016). CISD1 inhibits ferroptosis by protection against mitochondrial lipid peroxidation. *Biochem. Biophys. Res. Commun.* 478 838–844. 10.1016/j.bbrc.2016.08.034 27510639

[B24] Zeindler J. Angehrn F. Droeser R. Däster S. Piscuoglio S. Ng C. K. Y. (2019). Infiltration by myeloperoxidase-positive neutrophils is an independent prognostic factor in breast cancer. *Breast Cancer Res. Treat.* 177 581–589. 10.1007/s10549-019-05336-3 31267330

[B25] Zhou N. Bao J. (2020). FerrDb: a manually curated resource for regulators and markers of ferroptosis and ferroptosis-disease associations. *Database* 2020:baaa021. 10.1093/database/baaa021 32219413 PMC7100629

