# Supplementary figures and images for: A Novel Ferroptosis-Related Gene Signature for Overall Survival Prediction in Patients With Breast Cancer
Source: Front Cell Dev Biol. 2021 Jun 17;9:670184. doi: 10.3389/fcell.2021.670184 (PMC8247647; doi:10.3389/fcell.2021.670184)

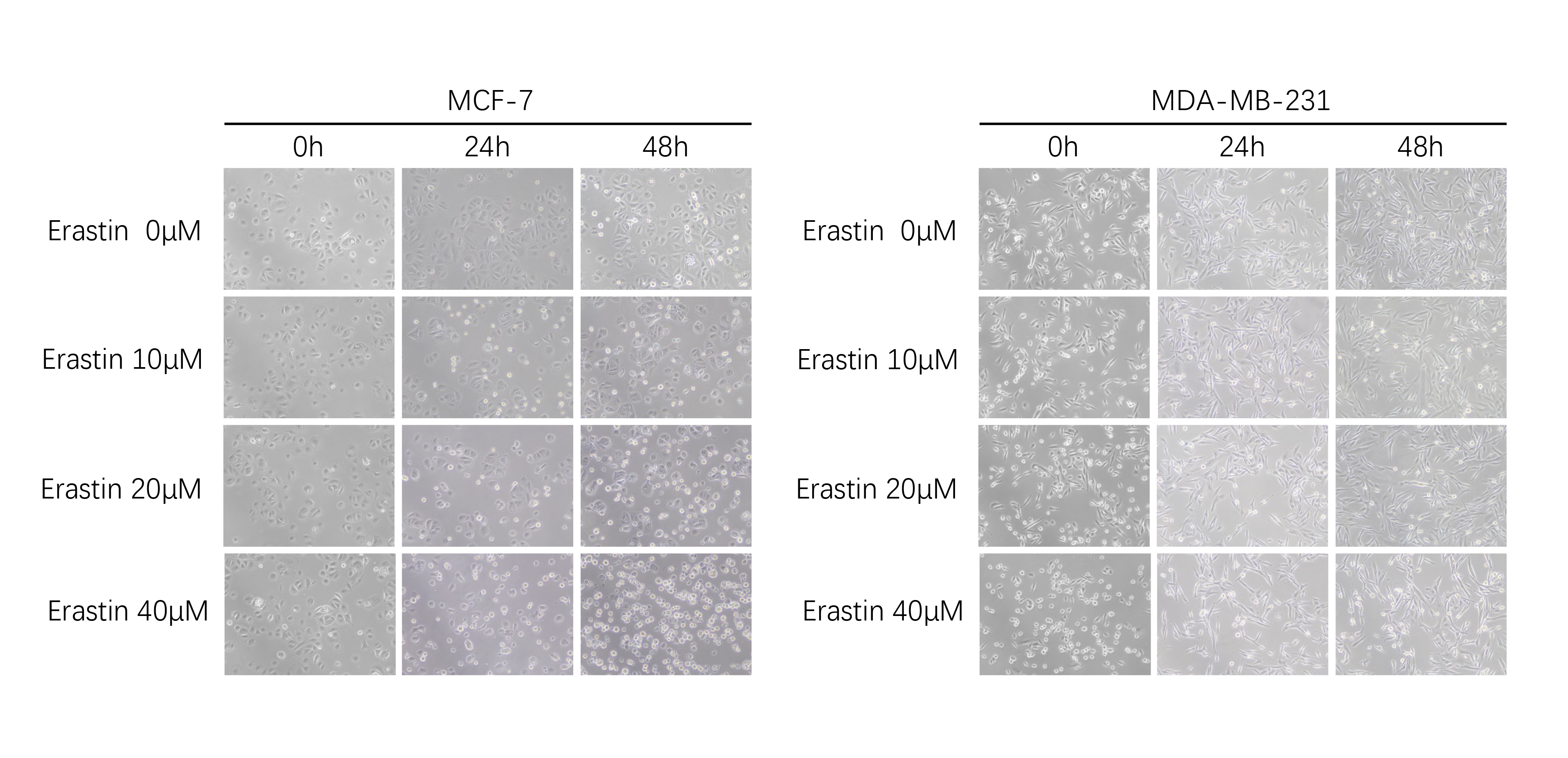

Supplement: Supplementary Figure 1 — The images about MCF7 cell viability and MDA-MB-231 treated by erastin. [file Image_1.TIF]

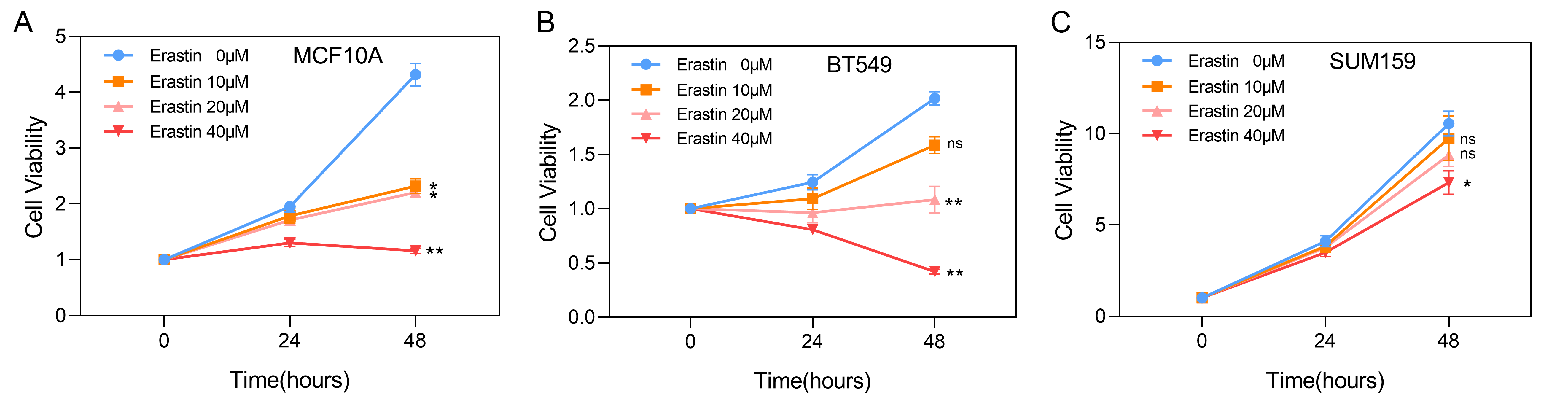

Supplement: Supplementary Figure 2 — The cell viability of MCF-10A (A), BT-549 (B), and SUM-159 (C) treated by erastin was tested by CCK8 assay. [file Image_2.TIF]

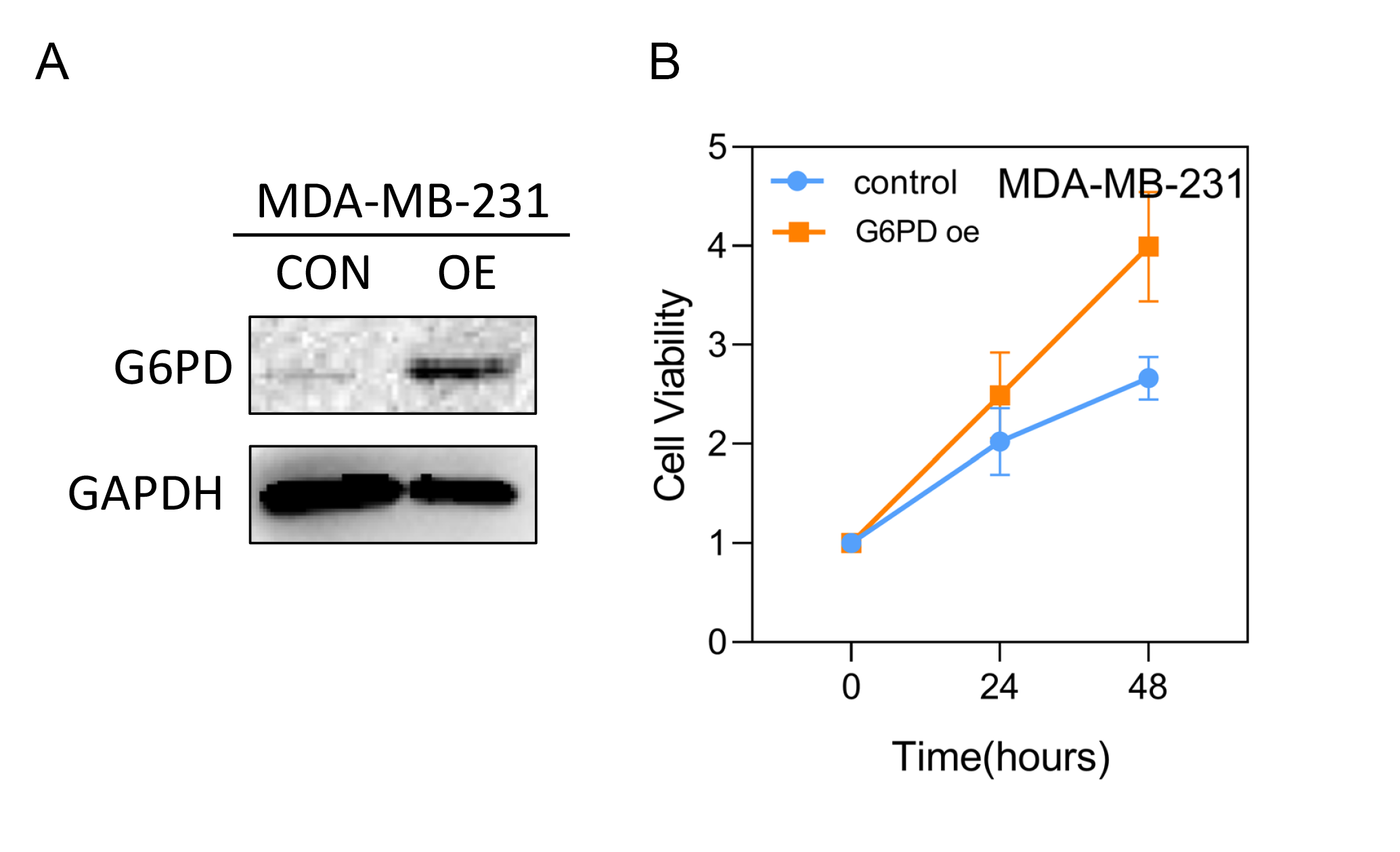

Supplement: Supplementary Figure 3 — The transfection of overexpression plasmid G6PD in MDA-MB-231 and the cell viability test. (A) The transfection efficiency was tested by western blot. (B) The viability of MDA-MB-231 con and MDA-MB-231 overexpression G6PD were tested by CCK8 assay. [file Image_3.TIF]

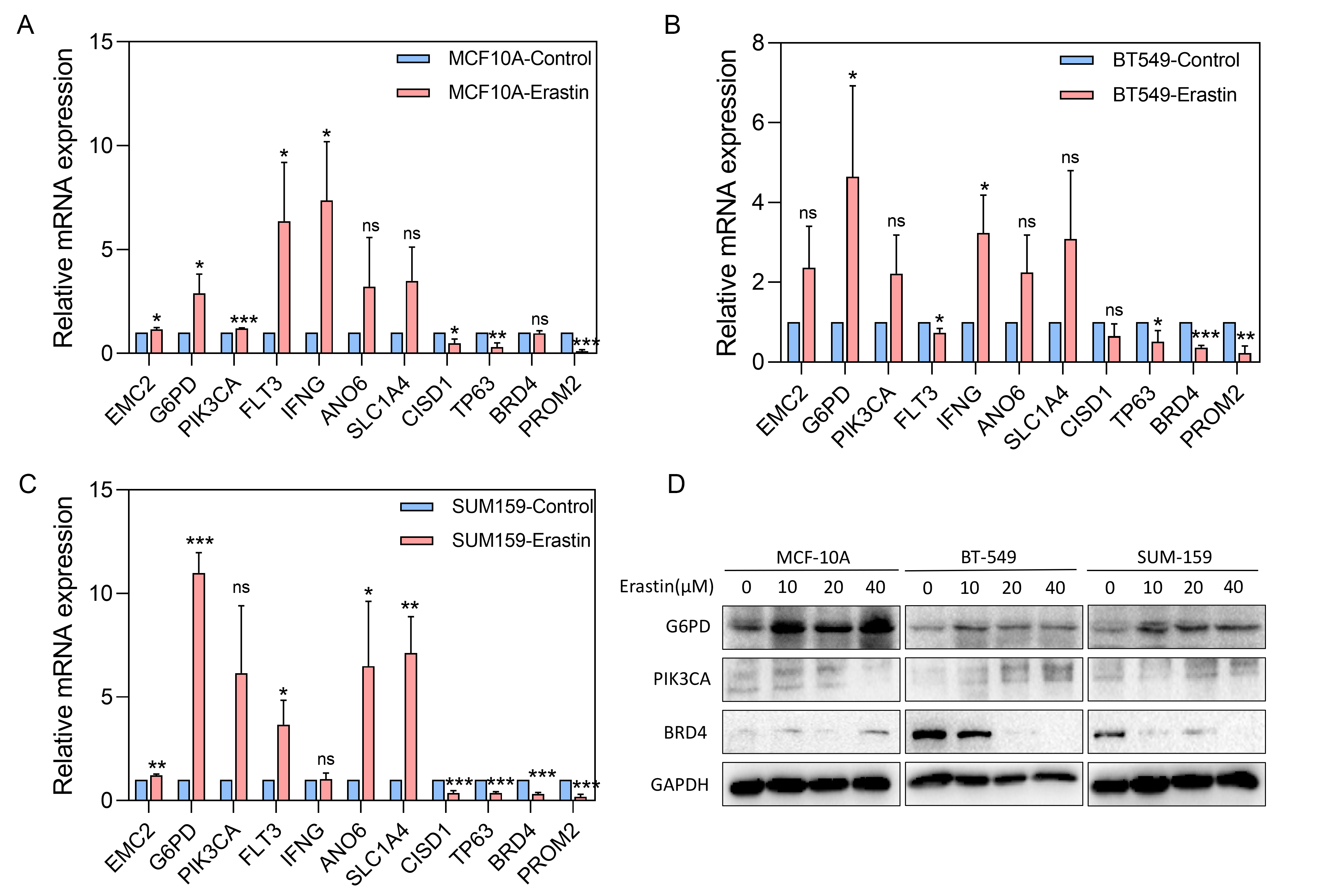

Supplement: Supplementary Figure 4 — Effects of ferroptosis inducer on expression levels of 11 core prognostic genes in breast cancer cell lines in vitro. (A–C) The expression changes of 11 core prognostic genes in MCF-10A (A), BT-549 (B), and SUM-159 (C) after treated with 20μM erastin were detected by real time-PCR. (D) The expression changes of G6PD, PIK3CA, and BRD4 genes in MCF-10A, BT-549, and SUM-159 after treated with erastin for 48 h was detected by western blot. [file Image_4.TIF]
