# Supplementary material for: A Novel Ferroptosis-Related Gene Signature for Overall Survival Prediction in Patients With Breast Cancer
Source: Front Cell Dev Biol. 2021 Jun 17;9:670184. doi: 10.3389/fcell.2021.670184 (PMC8247647; doi:10.3389/fcell.2021.670184)
Supplement: Supplementary file 7 [file Table_3.DOCX]

Supplementary Table 3. The sequences of primers for real-time PCR assays.

| Gene | Sequence |
| --- | --- |
| EMC2-F | TAGACTATGGTCGGGATGACTTG |
| EMC2-R | TGCCTGTTAATCGCTTGACTC |
| G6PD-F | ACCGCATCGACCACTACCT |
| G6PD-R | TGGGGCCGAAGATCCTGTT |
| PIK3CA-F | CCACGACCATCATCAGGTGAA |
| PIK3CA-R | CCTCACGGAGGCATTCTAAAGT |
| FLT3-F | AGGGACAGTGTACGAAGCTG |
| FLT3-R | GCTGTGCTTAAAGACCCAGAG |
| IFNG-F | TCGGTAACTGACTTGAATGTCCA |
| IFNG-R | TCGCTTCCCTGTTTTAGCTGC |
| ANO6-F | AAATTGCCTCTGAAACCCAATGA |
| ANO6-R | GCTTTCGTCTACACTGAGGACTT |
| SLC1A4-F | TGTTTGCTCTGGTGTTAGGAGT |
| SLC1A4-R  CISD1-F  CISD1-R  TP63-F  TP63-R  BRD4-F  BRD4-R  PROM2-F  PROM2-R | CGCCTCGTTGAGGGAATTGAA  GATCGCAGCAGTTACCATTGC  GCATGTACTATCTTGGGGTTGTC  CCACCTGGACGTATTCCACTG  TCGAATCAAATGACTAGGAGGGG  ACCTCCAACCCTAACAAGCC  TTTCCATAGTGTCTTGAGCACC  CGTGGCACAGCAATTCTCC  CAGCTCTACCACTGTAGCATTC |
