# Supplementary material for: A Novel Ferroptosis-Related Gene Signature for Overall Survival Prediction in Patients With Breast Cancer
Source: Front Cell Dev Biol. 2021 Jun 17;9:670184. doi: 10.3389/fcell.2021.670184 (PMC8247647; doi:10.3389/fcell.2021.670184)
Supplement: Supplementary file 10 [file Presentation_1.PPTX]

## Slide 1
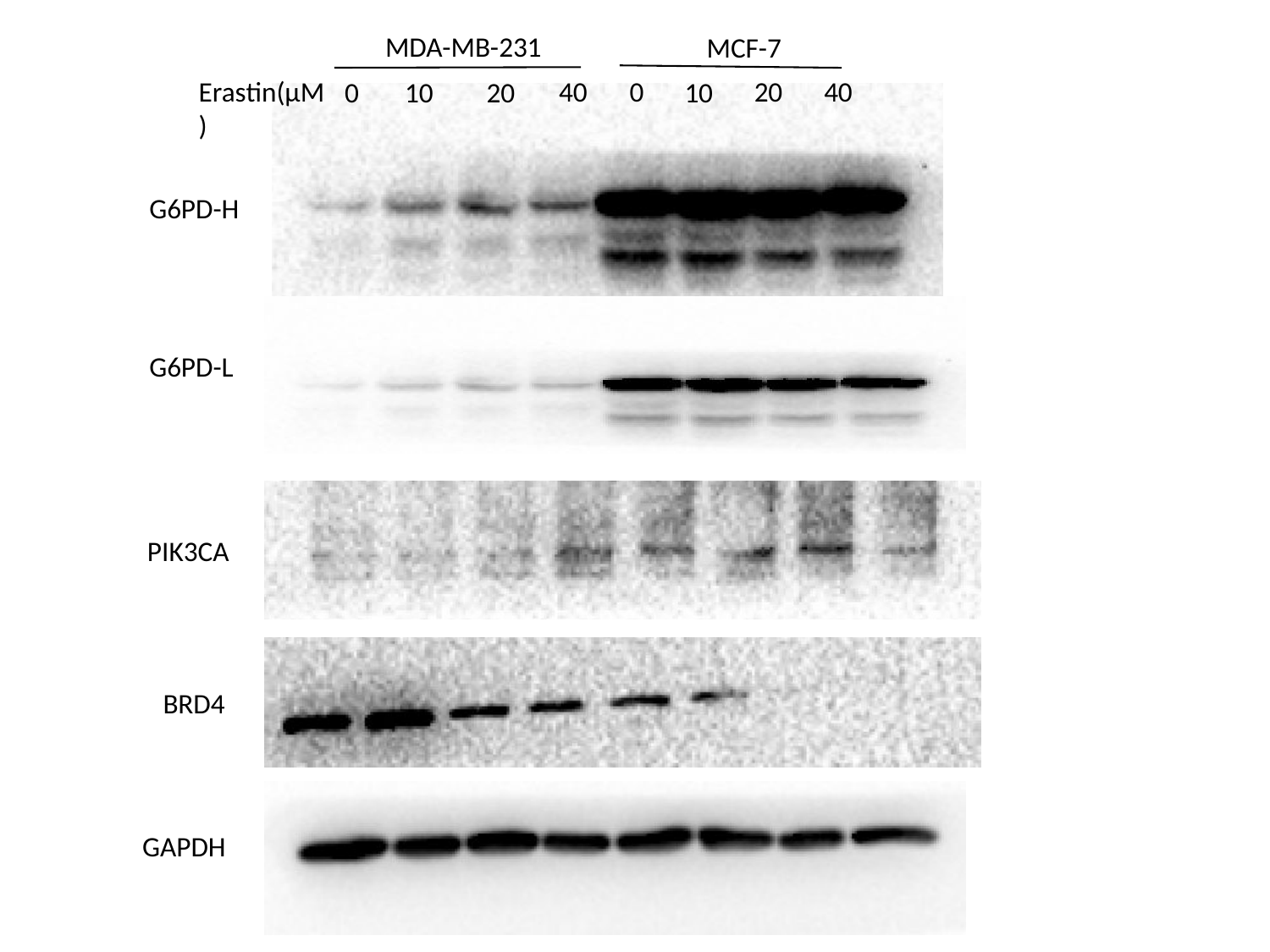

MDA-MB-231
MCF-7
20
Erastin(μM)
40
0
40
0
10
20
10
G6PD-H
G6PD-L
PIK3CA
BRD4
GAPDH

## Slide 2
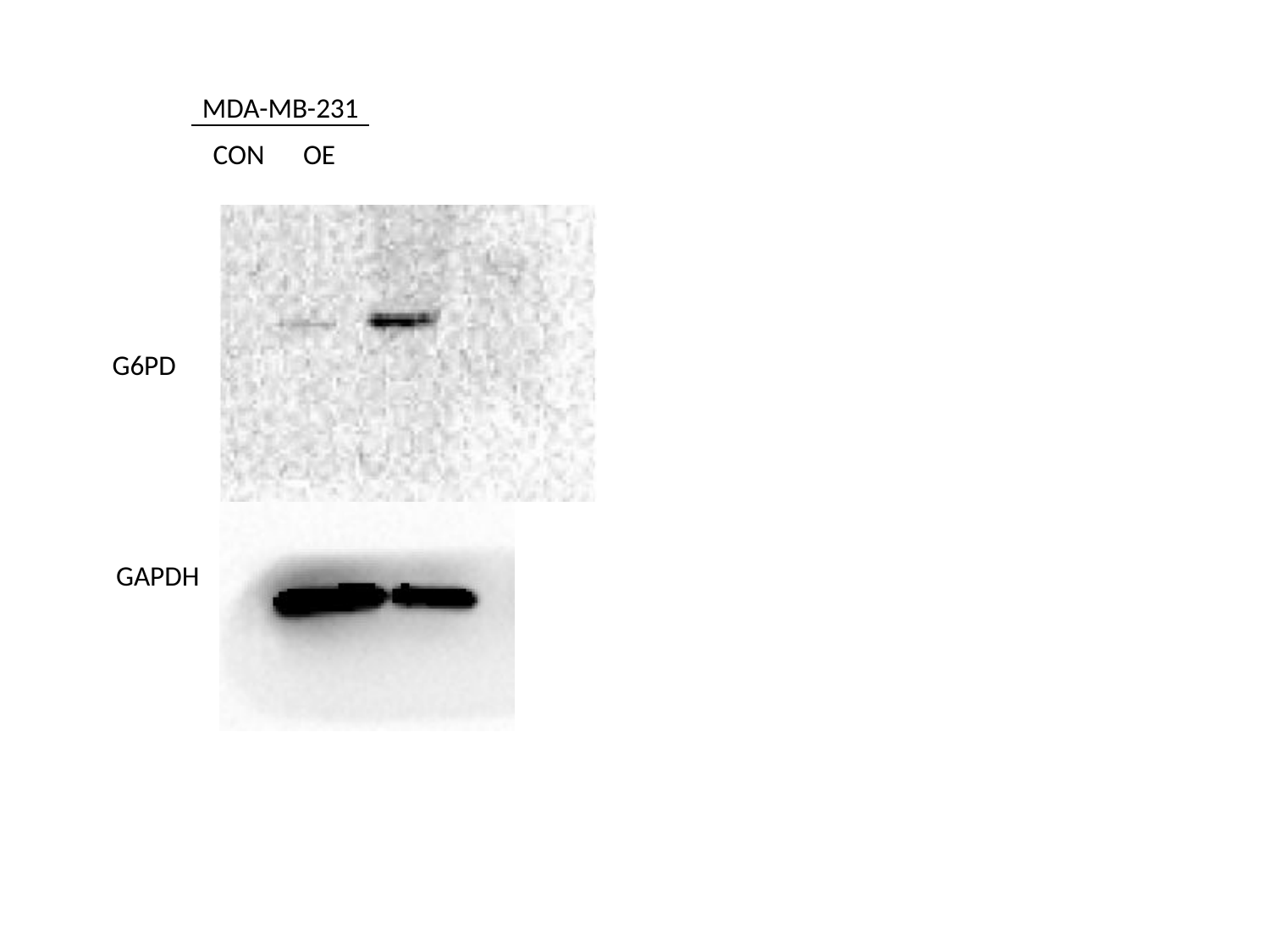

MDA-MB-231
CON
OE
G6PD
GAPDH

## Slide 3
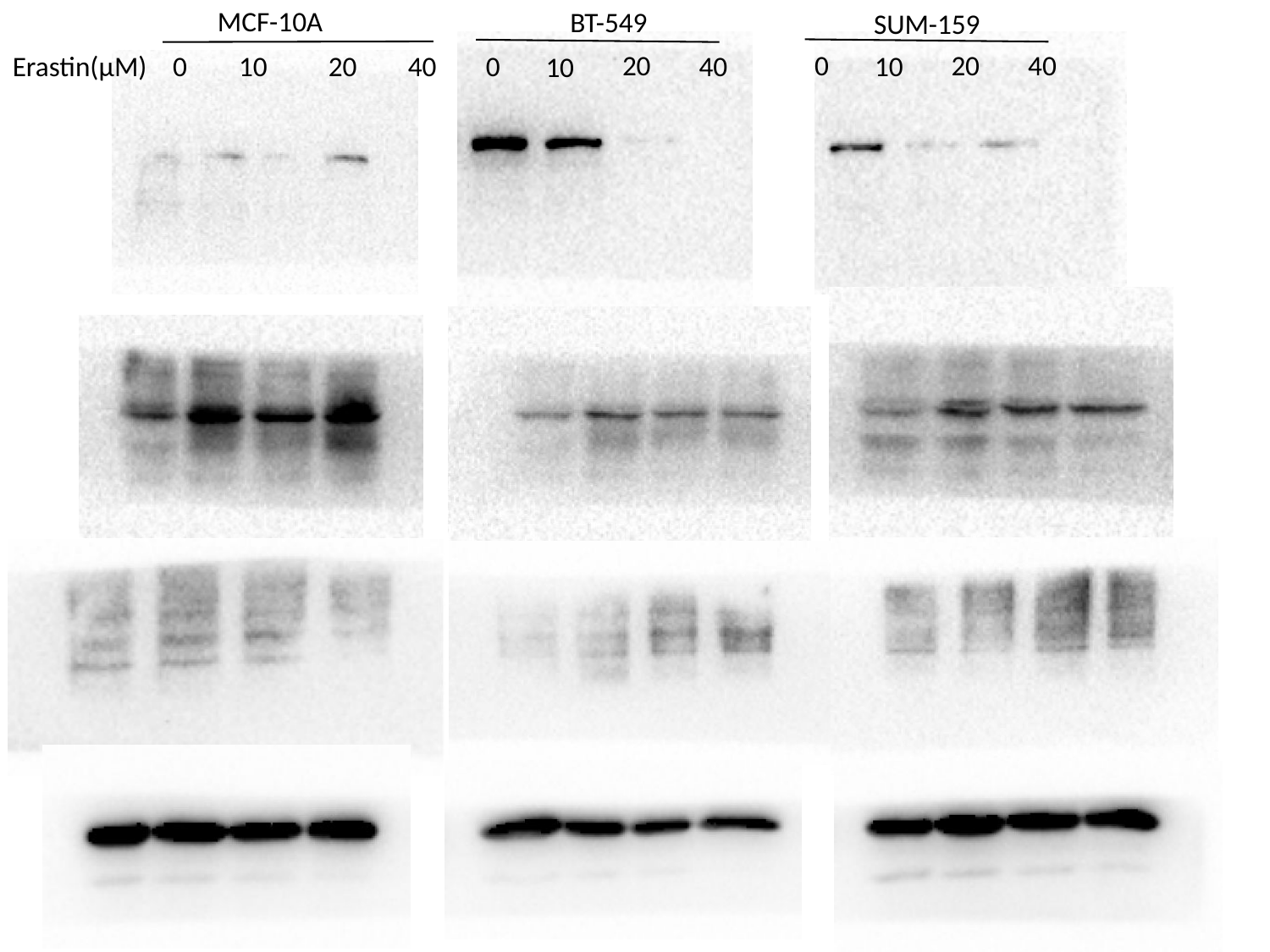

SUM-159
MCF-10A
BT-549
20
Erastin(μM)
40
0
40
0
10
20
10
20
0
40
10
